# Gender-related differences in the prevalence of cardiovascular disease risk factors and their correlates in urban Tanzania

**DOI:** 10.1186/1471-2261-9-30

**Published:** 2009-07-17

**Authors:** Marina A Njelekela, Rose Mpembeni, Alfa Muhihi, Nuru L Mligiliche, Donna Spiegelman, Ellen Hertzmark, Enju Liu, Julia L Finkelstein, Wafaie W Fawzi, Walter C Willett, Jacob Mtabaji

**Affiliations:** 1 Department of Physiology, Muhimbili University of Health and Allied Sciences, PO Box 65001, Dar es Salaam, Tanzania; 2 Department of Epidemiology and Biostatistics, Muhimbili University of Health and Allied Sciences, PO Box 65001, Dar es Salaam, Tanzania; 3 Department of Anatomy and Physiology, School of Medicine, Weill Cornell University, Qatar Campus, Doha, Qatar; 4 Department of Epidemiology, Harvard School of Public Health, Boston, Massachusetts, USA; 5 Department of Biostatistics, Harvard School of Public Health, Boston, Massachusetts, USA; 6 Department of Nutrition, Harvard School of Public Health, Boston, Massachusetts, USA; 7 Department of Global Health and Population, Harvard School of Public Health, Boston, Massachusetts, USA; 8 Department of Physiology, Weill Bugando Medical University, Mwanza, Tanzania

## Abstract

**Background:**

Urban areas in Africa suffer a serious problem with dual burden of infectious diseases and emerging chronic diseases such as cardiovascular diseases (CVD) and diabetes which pose a serious threat to population health and health care resources. However in East Africa, there is limited literature in this research area. The objective of this study was to examine the prevalence of cardiovascular disease risk factors and their correlates among adults in Temeke, Dar es Salaam, Tanzania. Results of this study will help inform future research and potential preventive and therapeutic interventions against such chronic diseases.

**Methods:**

The study design was a cross sectional epidemiological study. A total of 209 participants aged between 44 and 66 years were included in the study. A structured questionnaire was used to evaluate socioeconomic and lifestyle characteristics. Blood samples were collected and analyzed to measure lipid profile and fasting glucose levels. Cardiovascular risk factors were defined using World Health Organization criteria.

**Results:**

The age-adjusted prevalence of obesity (BMI ≥ 30) was 13% and 35%, among men and women (p = 0.0003), respectively. The prevalence of abdominal obesity was 11% and 58% (p < 0.0001), and high WHR (men: >0.9, women: >0.85) was 51% and 73% (p = 0.002) for men and women respectively. Women had 4.3 times greater odds of obesity (95% CI: 1.9–10.1), 14.2–fold increased odds for abdominal adiposity (95% CI: 5.8–34.6), and 2.8 times greater odds of high waist-hip-ratio (95% CI: 1.4–5.7), compared to men. Women had more than three-fold greater odds of having metabolic syndrome (p = 0.001) compared to male counterparts, including abdominal obesity, low HDL-cholesterol, and high fasting blood glucose components. In contrast, female participants had 50% lower odds of having hypertension, compared to men (95%CI: 0.3–1.0). Among men, BMI and waist circumference were significantly correlated with blood pressure, triglycerides, total, LDL-, and HDL-cholesterol (BMI only), and fasting glucose; in contrast, only blood pressure was positively associated with BMI and waist circumference in women.

**Conclusion:**

The prevalence of CVD risk factors was high in this population, particularly among women. Health promotion, primary prevention, and health screening strategies are needed to reduce the burden of cardiovascular disease in Tanzania.

## Background

Traditionally, attention to health problems by researchers and policy makers in Sub-Saharan Africa (SSA) has focused on infectious diseases. However, changes in demographic and epidemiological determinants of health, particularly changes in lifestyle associated with urbanization, have resulted in an epidemiological and nutrition transition towards a greater prevalence of non-communicable diseases [1]. The dual burden of persistent infectious diseases such as HIV/AIDS, malaria, and tuberculosis, and emerging chronic diseases such as cardiovascular diseases and diabetes mellitus, poses a serious threat to population health and limited health care resources [2].

Overweight and obesity are leading risk factors for a number of chronic diseases, including cardiovascular disease (CVD), diabetes, and cancer. Obesity is a leading determinant of hypertension, dyslipidemia and diabetes mellitus [3]. Hypertension is the most common CVD risk factor worldwide, and one of the most important preventable risk factor for premature death [4].

Once considered a problem only in high-income countries, the prevalence of CVD risk factors is dramatically increasing in low- and middle-income African countries, particularly in urban areas [5,6]. For example, studies in Tanzania have reported high rates of hypertension in both urban and rural areas, particularly among the obese and the elderly [7]. The estimated prevalence of diabetes in SSA is about 1% in rural areas, 5 to 7% in urban areas, and between 8% and 13% in countries such as Uganda and South Africa [8,9]. In urban areas of Tanzania, the prevalence of diabetes mellitus has been reported to be 3.9% among men and 2.8% among women [10]. A generally favorable lipid profile (low total and LDL-cholesterol, and normal to high HDL-cholesterol) and low homocysteine values have been reported among the general population in Africa [11]. However, hyperlipidemia is becoming increasingly common. For example, studies from Tanzania have observed 25% prevalence of elevated serum total cholesterol (cholesterol >5.2 mmol/L) [7], and 15% prevalence of elevated triglycerides (TG ≥ 1.7 mmol/L), among adults over 35 years of age [12], with women affected more than men.

We conducted a cross-sectional assessment of the prevalence and correlates of CVD risk factors, among middle-aged men and women in Temeke District, Dar es Salaam, Tanzania. There is very limited literature in this research area, particularly from East Africa. This study will help inform future research and potential preventive and therapeutic interventions against such chronic diseases.

## Methods

### Study Design and Population

This study was conducted in five administrative wards in Temeke, an urban district of Dar es Salaam, Tanzania, with a population of 768,451 (94% urban) (Tanzania National Bureau of Statistics 2002). A total of five administrative wards (of 24) in Temeke were randomly selected for this study, namely: Mbagala, Mjimwema, Kimbiji, Kigamboni and Vituka.

A total of 250 adult (44–66 years) residents, were randomly selected from a list stratified by gender, and invited to participate in the study. Eligibility criteria were identified *a priori*, due to the increased risk of CVD in middle-aged adults, importance of including both pre- and post-menopausal women, and to facilitate comparison with two previous studies conducted in the Temeke district [7,10]. A total of 115 men and 94 women were enrolled in the study; the refusal rate was 16.4%, mainly due to concerns regarding HIV testing of blood samples (verbal communication).

Informed consent was obtained from all participants. The research protocol was approved by the Research and Publications Committee of Muhimbili University College of Health Sciences and the Institutional Review Board of the Harvard School of Public Health.

### Assessment of Covariates

All measurements were conducted by a trained physician and one nurse. Questionnaires were administered by two trained research assistants. Anthropometric measurements, including height, weight, waist and hip circumferences, were obtained using standardized procedures. Body mass index (BMI) was calculated as weight divided by height squared (kg/m^2^), and categorized as normal (<25.0), overweight (25.0–<30.0), and obese (≥30.0) [13]. We did not use the underweight category in this population as none of the participants had BMI less than 18.5 kg/m^2^. Abdominal obesity was defined as waist circumference ≥ 102 centimeters in men and ≥ 88 centimeters in women [13]. Waist-to-hip ratio was calculated by dividing waist circumference by hip circumference. High waist circumference was defined as ≥ 0.9 in men and ≥ 0.85 in women [13].

Blood pressure measurements were conducted in the morning upon participant's arrival to the study sites. Participants were advised to not eat or drink anything before measurements, and we ensured that the participants had not consumed alcohol or coffee before coming to the study that may affect blood pressure measurements. Blood pressure was measured using a standardized digital blood pressure measuring machine (Omron Digital HEM-907, Tokyo, Japan). Three blood pressure readings were taken on the left upper arm with the participant in a seated position after at least 5 to 10 minutes of rest. The average of the three readings was used in this analysis. Severe hypertension was defined as ≥ 160/95 mmHg, in accordance with the WHO/ISH Classification of Hypertension [14].

Blood was collected by a qualified phlebotomist. Blood samples were separated within 6 to 8 hours of specimen collection and stored (≤-80°C) for four weeks, and batch tested by a senior technician; instruments were calibrated daily based on standardized procedures. The laboratory participates in the College of American Pathologists proficiency testing programs where three general chemistry panels including lipids and two calibration verification panels are taken annually.

Total cholesterol, triglycerides, and HDL-cholesterol levels were measured using Cobas Integra 400 analyzer (Roche Diagnostics). High cholesterol was defined as ≥ 6.2 mmol/L (240 mg/dl) [15]. LDL-cholesterol was estimated using the Friedewald formula [i.e., LDL = total cholesterol - HDL - (TG/5)] [16]. High LDL-cholesterol was defined as ≥ 3.8 mmol/L; low HDL-cholesterol was defined as < 1.0 mmol/L for men and < 1.3 mmol/L for women [15]. High triglyceride levels were defined as ≥ 1.7 mmol/L [15]. The atherogenic index was defined as the ratio between total cholesterol and HDL-cholesterol [15]; in this study elevated atherogenic index was defined as a ratio ≥ 5.

Participants were diagnosed with diabetes mellitus if they had fasting blood glucose level of ≥ 7.0 mmol/L, reported a history of diabetes mellitus, or were currently receiving treatment for diabetes (i.e, insulin or oral hypoglycemic agents) [17]. Participants were classified as having metabolic syndrome if they satisfied at least 3 of the following 5 criteria: elevated waist circumference (men: >102 cm, women: >88 cm), blood pressure (men: ≥ 130 mmHg, women: ≥ 85 mmHg), fasting blood glucose (≥ 6.1 mmol/L), and triglycerides (≥ 1.7 mmol/L), and lower HDL-cholesterol (men: < 1.0 mmol/L, women: < 1.3 mmol/L) levels [15].

We evaluated participant socioeconomic status using a structured questionnaire. Total monthly income in Tanzanian shillings (Tshs) was categorized into three levels, according to government minimum wage in 2006, or 50,000 Tshs (approximately 40 USD): ≥ 50,000 Tshs, > 50,000 to 80,000 Tshs, and > 80,000 Tshs. Level of education was defined as: none (no formal education), primary education (≥ 7 years), and higher education (> 7 years, including vocational training, secondary, high school, college, university, and post-graduate education). Socioeconomic indicators were evaluated using the Filmer-Pritchett wealth index [18], and dichotomized at the 75^th ^percentile as "poor" and "rich".

Dietary intake was assessed *via *a food frequency questionnaire [19]. This questionnaire consisted of 85 foods; for each item, type, frequency, and portion size were assessed.

The Sub-Saharan Africa Activity Questionnaire (SSAAQ) is interviewer administered questionnaire that evaluates occupation, cycling, walking as a form of transportation, and leisure-time activity, over the last 12 months, to account for seasonal variations in the level of physical activity [20]. Frequency, duration, and intensity were measured for each activity reported. Self-reported intensity of physical activities was defined as none, light, moderate, and intense. Frequency and duration of leisure-time physical activities that were performed at least six times over the past year were also assessed. Past month leisure-time physical activity was also recorded for the purpose of comparison with habitual leisure-time physical activity.

Alcohol intake was evaluated using a food frequency questionnaire [19] and classified as: no alcohol intake, moderate (1–2 drinks per day or per session) or high (> 2 drinks per day or per session). Units of alcohol intake were defined according to standardized criteria for beer (341 mL or 12 oz.; 5% alcohol), wine (142 mL or 5 oz; 12% alcohol), and hard liquor (43 mL or 1.5 oz.; 40% alcohol) [21]. Tobacco use was categorized as never (no history of cigarette smoking or other types of tobacco use), current (≥ 1 cigarette per day for ≥ 1 month) and former (ever smoked or used tobacco products) smoker [22].

### Data Analysis

The distribution of categorical socio-demographic, behavioral and correlates of cardiovascular disease (CVD) risk factors was compared across gender by using the χ^2^-test for the difference between proportions. For continuous CVD risk factors, crude comparisons were done with the Wilcoxon test. Adjusted means were calculated using Generalized Estimation Equations (GEE) models with the identity link function for normally distributed outcomes [23]. Covariates included age (<50, 50–54, 55–59, ≥60 years), occupation (not employed, public/private institutions, self employed/business, farmers), wealth factor (poor, rich), income (low, median, high), education (high, primary, no education), and physical activity (<26, 26–37, >37 MET-hours/day).

Spearman correlation coefficients (*r*) were calculated for blood pressure, lipid profile, and glucose levels with BMI and waist circumference. For categorical CVD risk factors, we used logistic regression to estimate the odds ratio after adjusting for potential confounders [24].

In all the analyses, a p-value of < 0.05 was considered statistically significant. Data analysis was carried out with the Statistical Analysis System (SAS) Software, version 9.1.

## Results

### Socio-demographic and Behavioral Characteristics

A total of 115 men and 94 women, aged 44 to 66 years, were included in this analysis. Participants were residents of five districts in Temeke: 37 from Mbagala, 26 from Mjimwema, 36 from Kimbiji, 57 from Kigamboni, and 53 from Vituka. The socio-demographic and clinical characteristics of participants are summarized in Table 1. Women were significantly more likely to have poorer indicators for education (p = 0.005), income (p = 0.005), and occupation (p = 0.0001), compared to men. Men were significantly older (p < 0.001) and had higher rates of smoking (p < 0.0001) and alcohol intake (p = 0.003).

**Table 1:** Gender differences in demographic and behavioral characteristics of participants

	Male^1 ^(n = 115)	Female^1 ^(n = 94)	**P-value**^2^
**Age **(mean, years)	53	51	<0.001
**Education**			
Higher Education (> 7 years)	31	17	
Primary Education (≤ 7 years)	60	61	0.005
No Education	9	22	
**Administrative ward of residence**			
Mbagala	18.3	17.0	
Mjimwema	9.6	16.0	0.43
Kimbiji	20.9	12.8	
Kigamboni	26.1	28.7	
Vituka	25.2	25.5	
**Individual income per month**^3^			
Low (≤50,000 Tshs/m)	56	71	
Medium (>50,000–80,000 Tshs/m)	23	6	0.005
High (>80,000 Tshs/m)	21	22	
**Occupation**			
Not Employed	8	31	
Public/Private Institutions	14	12	0.0001
Self Employed/Business	36	36	
Farmers	42	21	
**Current Smoker**			
Yes	23	3	<0.0001
No	77	97	
**Current alcohol consumption**			
Yes	37	18	0.003
No	63	82	
**High alcohol intake (≥4 servings/day)**			
Yes	31	12	0.001
No	69	88	
**Physical activity**^4 ^**(#MET-hours/day)**			
<26	34	33	
26–37	39	26	
>37	27	41	0.06

^1^Values are given as percentages (%) unless indicated

^2^Significance test: χ^2^-test for category variables and Kruskal-Wallis test for continuous variables

^3^1300 Tshs = 1 US dollars

^4^Tertile cut-points was used to define physical activity categories

### Gender Differences in Risk Factors for Cardiovascular Disease

Women had significantly higher mean BMI, waist and hip circumference, and waist-to-height ratio, and lower systolic and diastolic blood pressure level, compared to men [see Additional file 1]. Findings remained statistically significant after adjusting for age, occupation, wealth factor, income, education, and physical activity. In contrast, triglycerides, total, LDL-, and HDL-cholesterol, and fasting blood glucose levels did not significantly differ by gender.

In logistic regression analyses, the odds of overweight, severe hypertension (≥160/95 mmHg), hypercholesterolemia (≥6.2 mmol/L), elevated LDL-cholesterol (≥3.8 mmol/L), and diabetes did not significantly differ by gender [see Additional file 2]. After adjusting for age, occupation, wealth factor, income, education, and physical activity, women had 4.3 times greater odds of being obese, 14.2 times greater odds of abdominal obesity, and 2.8-fold greater odds of having a high WHR, compared to men. Women had more than three-fold greater odds of having metabolic syndrome (p = 0.001); in particular, abdominal obesity, low HDL-cholesterol, and high fasting blood glucose components of metabolic syndrome, compared to their male counterparts. In contrast, women had 50% lower odds of having high blood pressure, compared to men.

**Figure 1:**
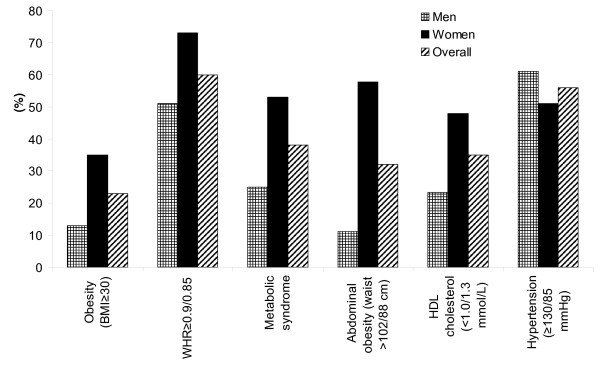
**Prevalence of CVD risk factors by gender**. (All P-values for gender differences < 0.05, after adjusting for age).

Age-adjusted prevalence of CVD risk factors by gender are presented in Figure 1. A total of 33% of participants were overweight, 23% were obese, and almost one-third had abdominal obesity. Hypertension (57%), including severe hypertension (30%) was common, and 84% of participants had hypertriglyceridemia. Although only 6% of participants had diabetes mellitus, 38% satisfied at least three of the criteria for metabolic syndrome. Among women, the prevalence of obesity, abdominal obesity, higher WHR, metabolic syndrome, and elevated HDL-cholesterol were significantly greater, compared to men.

### Correlates of CVD Risk Factors

Among men, body mass index and waist circumference were significantly correlated with CVD risk factors including total and LDL-cholesterol, triglycerides, systolic and diastolic blood pressure, and fasting blood glucose (p < 0.05) [see Additional file 3]. In contrast, among women, body mass index and waist circumference were not significantly associated with most CVD risk factors; BMI was correlated with SBP and DBP, and waist circumference was associated with DBP (p < 0.05).

In an analysis of the overall sample, each unit increase in BMI was associated with a 10% increase the odds of hypertension (Odds ratio [OR] 1.1, 95% CI: 1.1–1.2, p < 0.0001), and each 0.1 unit increase in WHR was associated with a 70% increase in the odds of hypertension (OR: 1.7, 95% CI: 1.0–2.8, p = 0.04).

## Discussion

In this study among adults in an urban East African setting, we noted a high prevalence of cardiovascular disease risk factors, including obesity, abdominal obesity, hypertension, dyslipidemia, diabetes mellitus, and metabolic syndrome. Several gender disparities in CVD risk factors were observed, with particularly high levels of obesity, abdominal obesity, and metabolic syndrome among women.

The prevalence of excess body weight was substantial in this study, particularly among women. This is consistent with findings from a previous study in Tanzania, which noted a high proportion of overweight among women aged 47 to 57 years [7], and is consistent with other studies among women in urban areas in Cameroon and Kenya [5,6]. The high proportions of overweight and obesity among women in the urban areas of Dar es Salaam may be attributed to socio-cultural factors, namely gender-specific patterns of work activities, sedentary lifestyle [25], and cultural standard of physical attractiveness in African countries [26].

Despite a high prevalence of overweight and obesity in this study, 53% of women perceived their weight as normal; 15% of them were actually obese and 16% had no intention of losing weight. Thus, the importance of weight loss and maintenance for prevention of cardiovascular disease and overall health may need to be emphasized and promoted in this population. This assumes greater importance in light of increasing prevalence of obesity among women in urban African settings [27].

In this population we found an increasing trend of the prevalence of hypertension. Findings are consistent with previous studies in urban sub-Saharan African settings, and higher than previous reports in Tanzania. For example, in a study in Dar es Salaam in 2002, Bovet *et al *[28] reported a prevalence of hypertension (BP ≥ 140/90 mmHg or anti-hypertensive (anti-HT) drug use) of 27% and 30%, among men and women respectively, compared to 51% and 42% in the current study (2006). Similarly, the prevalence of severe hypertension (BP ≥ 160/95 mmHg or anti-HT drug use) in our study was 30% and 29% in men and women, compared to 13% and 18%, as reported in the previous study in 2002 [28]. The comparatively higher prevalence of hypertension in this study may be attributable to changes in dietary habits, socioeconomic status, sedentary lifestyle, and rates of obesity. However, we also observed 50% lower odds of hypertension among women compared to men, despite significantly higher rates of obesity. The comparatively lower rates of hypertension among women could be attributable to a protective effect of estrogen [29], since 65% of women were pre-menopausal, and lower rates of smoking compared to a previous study in Tanzania [28].

The association between increased body mass index and blood pressure is consistent with findings from previous studies in urban sub-Saharan African settings [10,11,27]. Obesity is associated with increased cardiac output, total blood volume, and arterial resistance, in part due to the increased metabolic demand of excess body weight [30]. In fact, at any given level of activity the cardiac workload is greater for obese individuals, with a corresponding increase in blood pressure [30]. Previous studies have shown a relationship between obesity and high blood pressure; a 10 kilogram increase in body weight has been associated with a 3.0 mmHg higher systolic blood pressure and 2.3 mmHg higher diastolic blood pressure [30].

In this study, women had significantly increased prevalence of obesity, but a reduced risk of hypertension. It is possible that increases in body fat mass may have different effects in women than in men, and that a greater degree of adiposity must be achieved in women to obtain a significant rise in blood pressure and an increase in a lipid risk profile comparable with that of men [31]. Nevertheless, overall findings suggest that obesity is an important risk factor for hypertension and severe hypertension in urban Tanzania; cardiovascular disease prevention efforts should target reductions in excess body weight, through weight reduction/maintenance strategies.

We also reported the overall prevalence of metabolic syndrome of 38% in this population; more than 53% of women had metabolic syndrome. We are not aware of other studies in urban African settings, which are directly comparable to our findings; however, a similar high prevalence of metabolic syndrome has been noted in adults in the United States [32]. Further research is warranted to examine the prevalence and components of metabolic syndrome in this population.

Some studies have shown a positive association of measures of adiposity with cholesterol, triglycerides, and LDL-cholesterol [33,34], while a study in Nigeria did not demonstrate such a relationship [35]. We observed a positive association between measure of obesity and components of the lipid profile in men, but not women. In particular, body mass index was associated with lower HDL-cholesterol, which is consistent with reports from previous studies [36,37]. In addition, we did not observe significant gender difference on CVD risk factors, for the adjusted means of weight, WHR, cholesterol levels, and fasting glucose in this study. This could be due to the fact that for statistically significant changes in weight, WHR, lipids and glucose to occur, much greater changes in lifestyle are required.

The prevalence of smoking has been reported to be increasing in Tanzania [38]. The prevalence of current smokers was lower in men (23%) and in women (3%) in our study compared to the 2002 study [38], 27% and 5%, respectively. This may be due to the fact that there was a wider age range in the previous study of 35–64 years compared to ours which was 44–66, suggesting that more young people were included in the previous survey. In both studies women smoked less than men. Although smoking rates were comparatively lower in this study population, smoking remains an important risk factor for hypertension and cardiovascular disease and more prevention/cessation programs are warranted in this population.

From the point of view of prevention of non-communicable diseases, weight control is an important priority in both men and women. An estimated 70 of women with central obesity were at risk of developing hypertension in this population. The detrimental effects of excess weight, particularly central adiposity, on blood pressure and lipid profile – important CVD risk factors – need to be addressed. Strategies should focus on a healthy diet, increased physical activity, and weight reduction and maintenance. Interventions to reduce weight gain are particularly warranted among women, and should address social, cultural, and gender-specific aspects of weight gain. The WHO and International Society of Hypertension risk prediction charts for assessment of cardiovascular risk factors for prevention and control of cardiovascular disease in low and middle-income countries needs to be implemented in this population [4].

Our study has several limitations. First, the cross-sectional sampling design does not allow inferences to be drawn with respect to the causal relationships among variables. Second, the study sample is only representative of adults residing in Temeke, and findings may therefore not be generalizable to Dar es Salaam and other urban African settings. Due to a limited sample size of 209, we cannot rule out that there may be additional gender-related differences that we did not have sufficient statistical power to detect. Despite these limitations, this study provides important data regarding the prevalence and correlates of gender-specific CVD risk factors among adults in an urban African setting.

## Conclusion

This study further adds strong evidence for the high prevalence of cardiovascular diseases risk factors in Tanzania, particularly among women. To date, no intervention addressing cardiovascular disease prevention has been implemented in Temeke District, and the rates of obesity and hypertension are rising steadily. Health promotion, primary prevention, and health screening strategies are needed to target hypertension, metabolic syndrome, diabetes, and obesity, and reduce the burden of cardiovascular disease in Tanzania.

## Competing interests

The authors declare that they have no competing interests.

## Authors' contributions

MN designed the study, carried out data collection, analysis and writing the manuscript. AM participated in data collection and contributed to the revision of the manuscript. RM, WF, EH, DS, WW, and EL participated in the analysis of the data, and reviewing for final submission. JM designed the study, participated in data collection and critically revised the manuscript. NM participated in drafting the proposal and revised the manuscript. All authors had read and approved the final manuscript.

## Pre-publication history

The pre-publication history for this paper can be accessed here:

http://www.biomedcentral.com/1471-2261/9/30/prepub

## Supplementary Material

Additional file 1 **Table 2. Gender differences in correlates of cardiovascular disease risk factors**. The table represents analysis of gender differences in correlates of cardiovascular diseases risk factors Click here for file

Additional file 2 **Table 3. Associations between cardiovascular disease risk factors and gender (comparing women to men)**. The table represents analysis of associations between cardiovascular disease risk factors and gender. Click here for file

Additional file 3 **Table 4. Correlations of body mass index and waist circumference with lipid profile, blood pressure, and fasting blood glucose**. The table represents the correlations of body mass index and waist circumference with lipid profile, blood pressure, and fasting blood glucose. Click here for file
