# Supplementary material for: Gender-related differences in the prevalence of cardiovascular disease risk factors and their correlates in urban Tanzania
Source: BMC Cardiovasc Disord. 2009 Jul 17;9:30. doi: 10.1186/1471-2261-9-30 (PMC2723083; doi:10.1186/1471-2261-9-30)
Supplement: Additional file 1 — Table 2. Gender differences in correlates of cardiovascular disease risk factors. The table represents analysis of gender differences in correlates of cardiovascular diseases risk factors [file 1471-2261-9-30-S1.doc]

**Table 2. Gender differences in correlates of cardiovascular disease risk factors**

| **Determinants** | **Unadjusted Means^1^** | | | **Adjusted Means^2^** | | |
| --- | --- | --- | --- | --- | --- | --- |
|  | **Men (n= 115)** | **Women (n= 94)** | **P-value** | **Men (n = 115)** | **Women (n = 94)** | **P-value** |
| **Weight** (kg) | 68.3 ± 14.0 | 68.3 ± 15.2 | 0.95 | 67.8 ± 1.3 | 69.2 ± 1.7 | 0.53 |
| **Body mass index** (kg/m^2^) | 25.0 ± 4.7 | 28.2 ± 6.5 | 0.0006 | 24.9 ± 0.5 | 28.3 ± 0.7 | 0.0002 |
| **Waist circumference** (cm) | 88.2 ± 11.4 | 93.0 ± 13.3 | 0.01 | 88.3 ± 1.1 | 93.2 ± 1.4 | 0.01 |
| **Hip circumference** (cm) | 98.7 ± 9.7 | 105.8 ± 13.0 | <0.0001 | 98.7 ± 1.0 | 106.0 ± 1.4 | 0.0001 |
| **Waist-to-hip ratio** | 0.89 ± 0.05 | 0.88 ± 0.07 | 0.01 | 0.89 ± 0.01 | 0.88 ± 0.01 | 0.16 |
| **Waist-to-height ratio** | 0.53 ± 0.07 | 0.60 ± 0.09 | <0.0001 | 0.54 ± 0.01 | 0.60 ± 0.01 | 0.007 |
|  |  |  |  |  |  |  |
| **Systolic blood pressure** (mmHg) | 142.7 ± 29.0 | 136.4 ± 28.3 | 0.05 | 144.1 ± 3.0 | 130.6 ± 3.1 | 0.03 |
| **Diastolic blood pressure** (mmHg) | 81.3 ± 18.3 | 78.6 ± 15.7 | 0.40 | 82.8 ± 1.9 | 76.4 ± 1.7 | 0.03 |
| **Heart rate** (b/pm) | 75.9 ± 13.2 | 80.2 ± 13.0 | 0.02 | 76.8 ± 1.3 | 79.2 ± 1.4 | 0.26 |
|  |  |  |  |  |  |  |
| **Total-cholesterol** (mmol/L) | 4.82 ± 1.13 | 5.16 ± 1.23 | 0.02 | 4.91 ± 0.10 | 5.03 ± 0.13 | 0.48 |
| **Triglycerides** (mmol/L) | 2.90 ± 1.61 | 2.83 ± 1.23 | 0.64 | 2.85 ± 0.13 | 2.89 ± 0.14 | 0.81 |
| **LDL-cholesterol** (mmol/L) | 2.96 ± 1.01 | 3.24 ± 1.18 | 0.06 | 3.05 ± 0.10 | 3.13 ± 0.12 | 0.63 |
| **HDL-cholesterol** (mmol/L) | 1.28 ± 0.45 | 1.35 ± 0.46 | 0.24 | 1.29 ± 0.04 | 1.32 ± 0.05 | 0.65 |
| **Atherogenic index^3^** | 4.48± 3.50 | 4.24 ± 1.67 | 0.75 | 4.40 ± 0.25 | 4.37 ± 0.22 | 0.94 |
|  |  |  |  |  |  |  |
| **Fasting blood glucose** (mmol/L) | 4.48 ± 1.82 | 4.97± 3.14 | 0.48 | 4.57 ± 0.18 | 4.88 ± 0.29 | 0.33 |

^1^ Unadjusted mean values are given as Mean ± SD; Kruskal-Wallis Test P-value

^2^ Adjusted mean values are given as Mean ± SE, the adjusted means were calculated at the mean level of each covariate; P-value for gender difference, adjusted for age (<50,50-54,55-59,≥60 years), occupation (not working, public/private institutions, self employed/business, farmers), wealth factor (poor, rich), income (low, median, high), education (high, primary, no education), and physical activity (<26,26-37,>37 MET-hours/day), from Generalized Estimation Equations (GEE) models using identity link for normally distributed outcomes

^3^Atherogenic Index=(Total Cholesterol/ HDL cholesterol)
