# Supplementary material for: Gender-related differences in the prevalence of cardiovascular disease risk factors and their correlates in urban Tanzania
Source: BMC Cardiovasc Disord. 2009 Jul 17;9:30. doi: 10.1186/1471-2261-9-30 (PMC2723083; doi:10.1186/1471-2261-9-30)
Supplement: Additional file 2 — Table 3. Associations between cardiovascular disease risk factors and gender (comparing women to men). The table represents analysis of associations between cardiovascular disease risk factors and gender. [file 1471-2261-9-30-S2.doc]

**Table 3. Associations between cardiovascular disease risk factors and gender (comparing women to men)^1^**

|  | |  | **Age-adjusted** | | **Multivariate-adjusted ^2^** | |
| --- | --- | --- | --- | --- | --- | --- |
|  | | **Male** | **Female** | | **Female** | |
|  | | Ref. | **OR** (95%CI) | **P-value** | **OR** (95%CI) | **P-value** |
| **Overweight** (25≤BMI<30) | | 1.00 | 0.8 (0.5-1.5) | 0.58 | 1.0 (0.5-2.0) | 0.97 |
| **Obese** (BMI≥30) | | 1.00 | 3.7 (1.8-7.4) | 0.0003 | 4.3 (1.9-10.1) | 0.0006 |
| **High Waist-to-hip ratio** (≥0.9/0.85) | | 1.00 | 2.1 (1.0-4.8) | 0.002 | 2.8 (1.4-5.7) | 0.005 |
| **Metabolic syndrome** | | 1.00 | 3.1 (1.7-5.7) | 0.0002 | 3.3 (1.6-6.8) | 0.001 |
|  | Abdominal obesity (WC>102/88 cm) | 1.00 | 10.9 (5.3-22.6) | <0.0001 | 14.2 (5.8-34.6) | <0.0001 |
|  | Triglycerides (≥1.7 mmol/L) | 1.00 | 1.1 (0.5-2.4) | 0.78 | 1.2 (0.5-2.9) | 0.65 |
|  | HDL-cholesterol (<1.0/1.3 mmol/L) | 1.00 | 3.0 (1.6-5.5) | 0.0004 | 3.4 (1.7-7.0) | 0.0007 |
|  | Blood pressure (≥130/85 mmHg) | 1.00 | 0.7 (0.4-1.3) | 0.24 | 0.5 (0.3-1.0) | 0.04 |
|  | Fasting glucose (≥6.1mmol/L) | 1.00 | 2.1 (0.7-5.8) | 0.17 | 2.6 (0.7-8.8) | 0.13 |
| **Severe hypertension** (≥160/95 mmHg) | | 1.00 | 0.9 (0.5-1.7) | 0.78 | 0.7 (0.4-1.5) | 0.38 |
| **Hypercholesterolemia** (≥6.2 mmol/L) | | 1.00 | 2.3 (0.9-5.9) | 0.09 | 1.9 (0.6-6.0) | 0.26 |
| **Elevated LDL** (≥3.8 mmol/L) | | 1.00 | 1.6 (0.0-3.0) | 0.18 | 1.5 (0.7-3.4) | 0.28 |
| **Diabetes^3^** | | 1.00 | 2.2 (0.7-7.2) | 0.18 | 2.0 (0.5-8.6) | 0.33 |

^1^ Logistic regression analyses

^2^ Adjusted for age (<50,50-54,55-59,≥60 years), occupation (not working, public/private institutions, self employed/business, farmers), wealth factor (poor, rich), income (low, median, high), education (high, primary, no education), and physical activity (<26,26-37,>37 MET-hours/day)

^3^ Diabetes diagnosis was based on FBG ≥ 7.0 mmol/L, reported a history of diabetes mellitus, or were currently receiving treatment for diabetes
