# Supplementary material for: Gender-related differences in the prevalence of cardiovascular disease risk factors and their correlates in urban Tanzania
Source: BMC Cardiovasc Disord. 2009 Jul 17;9:30. doi: 10.1186/1471-2261-9-30 (PMC2723083; doi:10.1186/1471-2261-9-30)
Supplement: Additional file 3 — Table 4. Correlations of body mass index and waist circumference with lipid profile, blood pressure, and fasting blood glucose. The table represents the correlations of body mass index and waist circumference with lipid profile, blood pressure, and fasting blood glucose. [file 1471-2261-9-30-S3.doc]

**Table 4. Correlations of body mass index and waist circumference with lipid profile, blood pressure, and fasting blood glucose**

| **Correlatess** | | **Men** | | **Women** | |
| --- | --- | --- | --- | --- | --- |
|  | | **r** | **P-value** | **r** | **P-value** |
| **Body mass index** (kg/m^2^) | |  |  |  |  |
|  | Total cholesterol (mmol/L) | 0.26 | 0.004 | 0.15 | 0.15 |
|  | Triglycerides (mmol/L) | 0.24 | 0.01 | 0.06 | 0.60 |
|  | LDL-cholesterol (mmol/L) | 0.31 | 0.001 | 0.16 | 0.14 |
|  | HDL-cholesterol (mmol/L) | -0.23 | 0.01 | -0.03 | 0.79 |
|  | Atherogenic Index^2^ | 0.04 | 0.67 | 0.09 | 0.42 |
|  | SBP (mmHg) | 0.30 | 0.001 | 0.21 | 0.04 |
|  | DBP (mmHg) | 0.31 | 0.001 | 0.33 | 0.001 |
|  | Fasting blood glucose (mmol/L) | 0.19 | 0.05 | 0.06 | 0.56 |
|  | |  |  |  |  |
| **Waist circumference** (cm) | |  |  |  |  |
|  | Total cholesterol (mmol/L) | 0.31 | 0.0007 | 0.15 | 0.16 |
|  | Triglycerides (mmol/L) | 0.24 | 0.009 | 0.08 | 0.42 |
|  | LDL-cholesterol (mmol/L) | 0.32 | 0.0006 | 0.14 | 0.18 |
|  | HDL-cholesterol (mmol/L) | -0.14 | 0.15 | -0.01 | 0.90 |
|  | Atherogenic index^2^ | 0.07 | 0.48 | 0.08 | 0.45 |
|  | SBP (mmHg) | 0.25 | 0.008 | 0.17 | 0.11 |
|  | DBP (mmHg) | 0.27 | 0.003 | 0.24 | 0.02 |
|  | Fasting blood glucose (mmol/L) | 0.18 | 0.05 | 0.14 | 0.19 |

^1^Values are Spearman correlation coefficients

^2^Atherogenic Index= (Total Cholesterol/ HDL cholesterol)
